# Large-Signal Linearity and High-Frequency Noise of Passivated AlGaN/GaN High-Electron Mobility Transistors

**DOI:** 10.3390/mi12010007

**Published:** 2020-12-24

**Authors:** Yu-Shyan Lin, Shin-Fu Lin

**Affiliations:** Department of Materials Science and Engineering, National Dong Hwa University, 1, Sec. 2, Da Hsueh Rd., Shou-Feng, Hualien 974, Taiwan; blackblue1982@gmail.com

**Keywords:** metallorganic chemical vapor deposition (MOCVD), passivation, HfO_2_, TiO_2_, GaN, high-electron mobility transistor (HEMT)

## Abstract

This study proposes AlGaN/GaN/silicon high-electron mobility transistors (HEMTs) grown by a metallorganic chemical vapor deposition (MOCVD) system. The large-signal linearity and high-frequency noise of HEMTs without and with different passivation layers are compared. The experimental data show that the addition of a TiO_2_ passivation layer to undoped AlGaN/GaN HEMT’s increases the value of the third-order intercept point (OIP3) by up to 70% at 2.4 GHz. Furthermore, the minimum noise figure (*NF*_min_) of the HEMT with TiO_2_ passivation is significantly reduced.

## 1. Introduction

Heterostructure field-effect transistor (HFET) technology has become essential in microwave communication systems [1,2]. III-nitride high-electron-mobility transistors (HEMTs) are of significant importance in the development of next-generation power applications [3,4,5]. The AlGaN/GaN heterostructure has the advantage of large electron velocity and high breakdown electric field. Furthermore, the polar properties of the AlGaN/GaN heterojunction allows the formation of two-dimensional electron gas (2DEG). Even without intentional doping, the 2DEG concentrations of the AlGaN/GaN HEMTs is as high as the order of 10^13^ cm^−2^.

Many passivation materials have been investigated to effectively passivate the surface of AlGaAs/InGaAs [6] and InAlAs/InGaAs/InP [7] HEMTs by using either a wet or dry process. The performance of AlGaN/GaN HEMT technology is limited by charge trapping effects. Consequently, various candidates for passivation has been attempted to neutralize the net surface charge arising from the combination of surface states and the polarized barrier [8,9,10,11,12,13,14,15,16,17,18]. Most works focus on the performance of the passivated AlGaN/GaN HEMTs at room temperature. The room-temperature characteristics of AlGaN/GaN HEMT with HfO_2_ and TiO_2_ passivation were reported [11]. However, AlGaN/GaN heterostructure is a promising material system for high-temperature electronics. HEMTs that can operate at high temperatures are helpful in broad extent of applications [13,19]. Consequently, the high-temperature characteristics of the passivated AlGaN/GaN HEMTs are measured herein. Furthermore, the linearity in power amplifier is important when we move towards the fifth generation (5 G) wireless systems. Increasing the linearity of HEMTs can supply many advantages at the system level. Consequently, the large-signal linearity of the passivated AlGaN/GaN HEMTs are also studied. To the best of the authors’ knowledge, there has not been a comparison of the large-signal linearity and noise figure for the AlGaN/GaN HEMTs with HfO_2_ and TiO_2_ passivation. Experimental results demonstrate the high-performance passivated HEMT with stable operation at elevated temperatures up to 420 K. The measured large-signal linearity and high-frequency noise of the passivated HEMT are better than for the identical geometry unpassivated HEMT.

## 2. Device Structure and Experiments

The studied devices were built on silicon substrate with epilayers that were grown by metal-organic chemical vapor deposition (MOCVD). The layer structure of the HEMT is as follows. First, a buffer was grown, followed by an undoped GaN. Then, 30 nm undoped Al_0.26_Ga_0.74_N layer was formed and capped by a 2 nm GaN layer.

**Figure 1:**
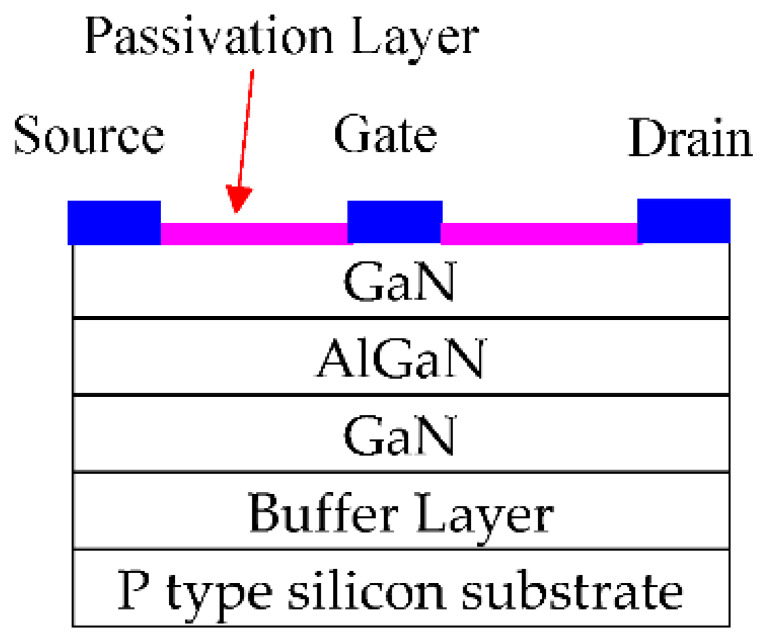
Cross section of the studied passivated AlGaN/GaN high-electron mobility transistor (HEMT).

Mesa etching was employed to achieve device isolation. Ti/Al/Au ohmic contacts for the source and drain electrodes were deposited. The gate metallization involved Ni, capped with Au. The HEMT without passivation is the reference HEMT. In our study, the HEMT with HfO_2_ passivation is referred to as HfO_2_-HEMT. The HEMTs with TiO_2_ passivation is referred to as TiO_2_-HEMT. The TiO_2_ film was sputtered in a sputtering system using a three-inch high-purity target of titanium dioxide in a mixture of argon and oxygen gas. HfO_2_ film was sputtered using hafnium dioxide. Figure 1 displays the layer structure of the studied HEMTs with passivation. The cross section of the passivated HEMTs was investigated by a transmission electron microscopy (TEM) (JEOL Co., Tokto, Japan). The probe station was fitted with a heated device stage. The DC characteristics of the HEMTs were measured with a Keithley 4200 semiconductor characterization system (Tektronix, Beaverton, OR, USA). The field-effect transistor had a gate length of 1 μm. The gate-to-drain spacing was 2 μm. The gate-to-source spacing was also 2 μm.

## 3. Results and Discussion

**Figure 2:**
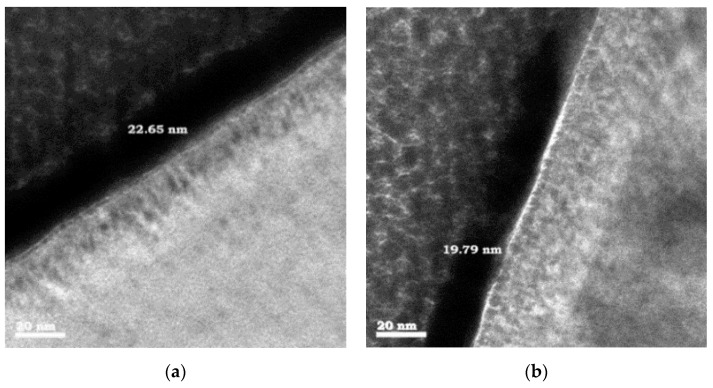
Cross-sectional TEM images of (**a**) HfO_2_-HEMT and (**b**) TiO_2_-HEMT.

TEM samples are examined in a JEM-2100F (JEOL Co., Japan) operating at an accelerating voltage of 200 kV. Figure 2 illustrates the TEM cross section of the HEMTs with HfO_2_ and TiO_2_. The thicknesses of HfO_2_ and TiO_2_ films are approximately 22.65 and 19.79 nm, respectively.

**Figure 3:**
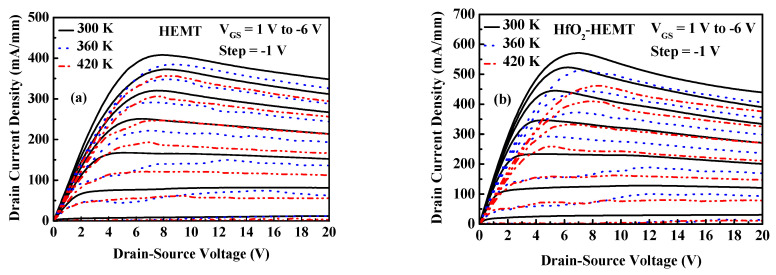

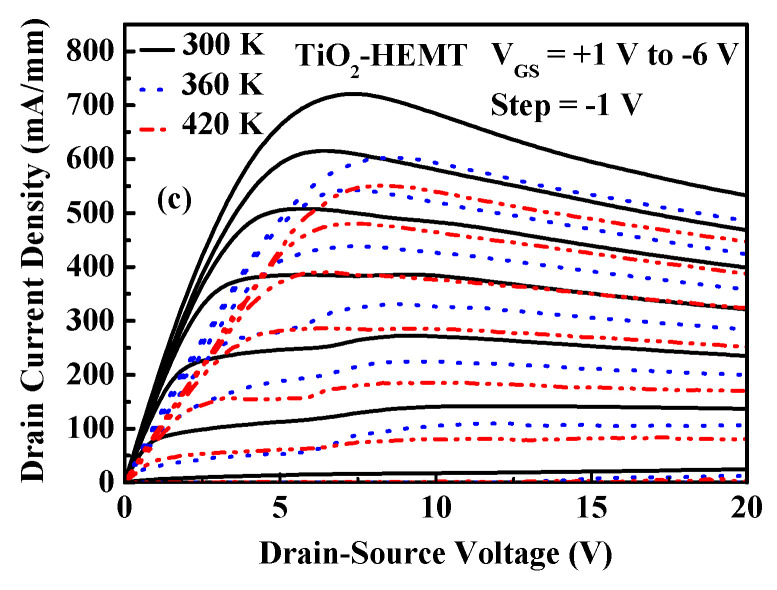
Family of drain-source output curves of (**a**) HEMT, (**b**) HfO_2_-HEMT, and (**c**) TiO_2_-HEMT at various temperatures.

**Figure 4:**
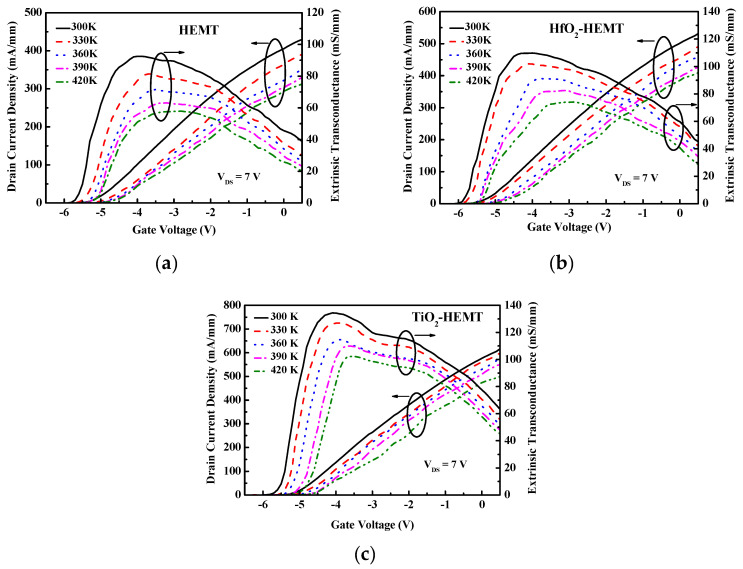
Extrinsic transconductance and drain current characteristics of (**a**) HEMT, (**b**) HfO_2_-HEMT, and (**c**) TiO_2_-HEMT at various temperatures.

**Figure 5:**
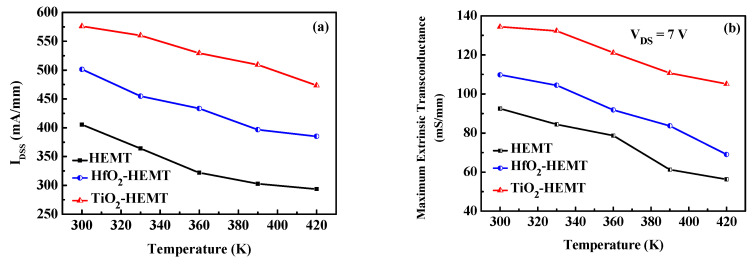
(**a**) *I*_DSS_ and (**b**) *g*_m,max_ of the studied HEMTs at various temperatures.

The unpassivated and passivated HEMTs are subjected to high-temperature testing. Figure 3 presents the drain currents (*I*_DS_) at different temperatures versus drain-to-source voltage (*V*_DS_). The DC measurements are taken as functions of temperatures over the range 300 to 420 K. Figure 4 shows the extrinsic transconductance (*g*_m_) and drain current versus gate-to-source voltage of the studied HEMTs at various temperatures. The gate voltage swing (GVS) is defined by the voltage range within which the *g*_m_ value deviates from its maximum value by 20%. The GVS value is increased from 1.7 V to 3.2 V at 300 K after TiO_2_ passivation. *I*_DS_ versus *V*_DS_ at pinch-off conditions and the threshold drain current characteristics at 300 K for the three HEMTs herein were studied [11]. Figure 5a plots drain current at *V*_GS_ = 0 V (*I*_DSS_) versus temperature of the studied HEMTs. Experimental results reveal that *I*_DSS_ values of the studied HEMTs are increased when the HEMTs are passivated. The increased drain current density is attributable to the increased sheet electron concentration after passivation [8,11]. The studied three HEMTs depicts good pinch-off characteristics at various temperatures. Increasing the temperature decreases *I*_DSS_. The falloff in drain current density at elevated temperatures result from the degradation of the electron mobility. Furthermore, the threshold voltage (*V*_th_) is extracted by linear extrapolation of the root of drain current against *V*_g_ curves. The values of *V*_th_ of the TiO_2_-HEMT are −5.5, −5.33, −5.2, −4.96, and −4.89 V at 300, 330, 360, 390, and 420 K, respectively. The magnitude of the *V*_th_ value is reduced at high temperature because of the decreased drain current density.

Figure 5b plots the maximum extrinsic transconductance (*g*_m,max_) versus temperature of the investigated HEMTs. When the temperature is increased, the maximum extrinsic transconductance varies in the same tendency as *I*_DSS_. At 420 K, the *g*_m,max_ values for HEMT, HfO_2_-HEMT, and TiO_2_-HEMT are 56.3, 69, and 105 mS/mm, respectively. Experimental results demonstrate the TiO_2_-HEMT perform well even at high temperatures.

**Figure 6:**
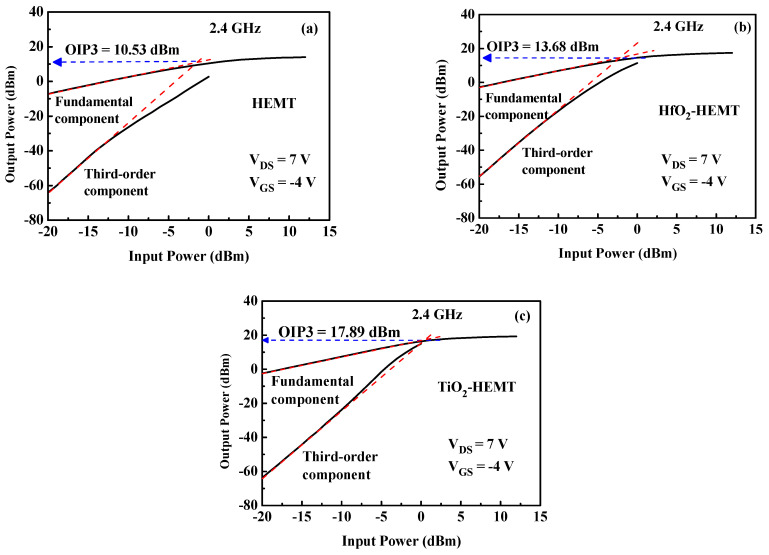
Fundamental output power and third-order intermodulation component of (**a**) HEMT, (**b**) HfO_2_-HEMT, and (**c**) TiO_2_-HEMT.

Two-tone intermodulation distortion is measured to demonstrate the large-signal linearity performance. Figure 6 shows the fundamental and third-order output powers versus input power of the studied devices. The red dashed lines are extrapolated to predict the intersection at the third-order intercept point (OIP3). The values of OIP3 are 10.5, 13.7, and 17.9 dBm, respectively. HfO_2_ passivation increases the OIP3 value by around 30% and TiO_2_ passivation increases it by 70%. The large-signal linearity of the HEMT is significantly improved when the HEMT is passivated by TiO_2_. The improved device linearity of the TiO_2_-HEMT is attributed to increased *g*_m,max_ [18] and GVS values [20].

**Figure 7:**
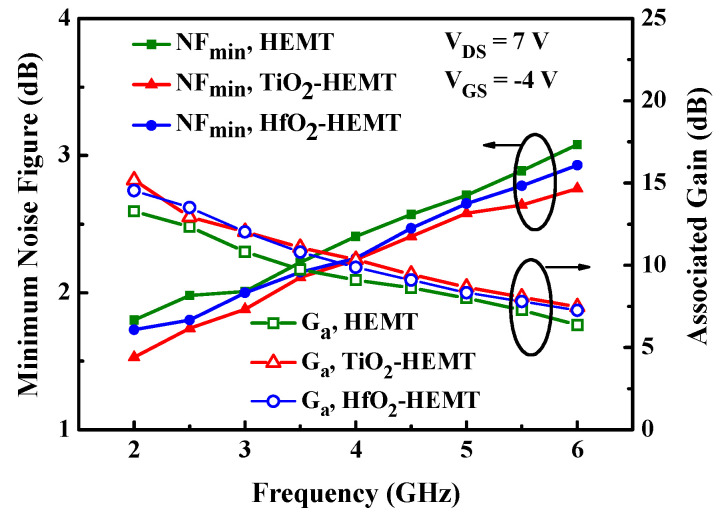
Minimum noise figure and associated gain of the studied (**a**) HEMT, (**b**) HfO_2_-HEMT, and TiO_2_-HEMT.

Noise figure is measured over the 2–6 GHz frequency range using an ATN NP5B noise parameter test set in conjunction with the HP-8510C network analyzer. Figure 7 shows the minimum noise figure (*NF*_min_) and associated power gain (*G*_a_) versus frequency for the studied HEMTs. Figure 7 reveals that the relationship between the noise and frequency is near linear. Quantitatively, *NF*_min_ is given by [21,22,23]

(1) $$NF_{\min}=1+2\pi fkC_{gs}\sqrt{\frac{R_{s}+R_{g}}{g_{m}}}$$

where *f* is frequency; *k* is the Fukui constant; *C_gs_* is the input gate-source capacitance; *R*_s_ is the source series resistance, and *R*_g_ is the gate series resistance. The *NF*_min_ values of HEMT, HfO_2_-HEMT, and TiO_2_-HEMT are 1.94 dB, 1.79 dB, and 1.68 dB. The TiO_2_-HEMT has the smallest *NF*_min_ of the three devices because it has the highest *g*_m_. Furthermore, the associate gain of the TiO_2_-HEMT is also improved.

## 4. Conclusions

AlGaN/GaN/silicon grown by MOCVD have been successfully fabricated and measured. The high-temperature characteristics of the proposed devices are investigated. TiO_2_-HEMT exhibits the best large-signal linearity of the studied devices. Furthermore, the *NF*_min_ value of TiO_2_-HEMT is smallest of the studied devices herein.
